# Delirium in adult and paediatric ICU patients: what is the way forward?

**DOI:** 10.1111/nicc.12629

**Published:** 2021-05-19

**Authors:** Erwin Ista, Peter Nydahl

**Affiliations:** ^1^ Department of Pediatric Surgery, Pediatric Intensive Care Erasmus MC—Sophia Children's Hospital Rotterdam The Netherlands; ^2^ Department of Internal Medicine, Nursing Science Erasmus MC Rotterdam The Netherlands; ^3^ Nursing Research; Department of Anaesthesiology and Intensive Care Medicine University Hospital Schleswig‐Holstein Kiel Germany

The theme of this issue of Nursing in Critical Care is delirium. Delirium is a serious, common complication in critical care, affecting up to 90% of adult patients and up to 74% of paediatric patients.^1^, ^2^ All nurses are familiar with the agitated, shouting patient, pulling out the tubes and lines, or the restless child, crying where nothing except drugs can calm them down. More serious—in terms of consequences—is the silent hypoactive delirium, often unnoticed, because patients seem to rest, they express no needs, do not cry for help, but may be lost in frightening experiences. Up to two thirds of delirium episodes of critically ill patients are not identified, because of several reasons: a lack of knowledge, lack of frequent assessments, inadequate staff numbers, lack of training, but more often, a lack of awareness of delirium.^3^, ^4^ More than 10 years ago, the British National Institute for Health and Care Excellence (NICE) guideline on delirium recommended: Think delirium^5^! This was, and is, the central recommendation of the delirium guideline. This is important because all healthcare professionals are busy, with many tasks and responsibilities, but if no one thinks about delirium, only the agitated patients will be noticed and, of note, only half of agitated patients actually have delirium.^6^ We now know that there is a dose–response relationship in delirium: the longer delirium is present, the more serious consequences of delirium, such as prolonged mechanical ventilation, longer stay in the Intensive Care Unit (ICU) and hospital, impaired cognition and rehabilitation, and higher mortality.^2^ Nevertheless, the implementation of delirium screening and prevention programs in adult and paediatric ICUs (PICU) is still challenging. This is especially so in critically ill children, in which regular monitoring of delirium with validated assessment tools was practiced in only 25% to 40% of PICUs.^7^, ^8^ Furthermore, there are many remaining research questions about the pharmacological and non‐pharmacological treatment of delirium.

So how can we identify patients at risk? It would be beneficial to be able to identify patients with a high risk of delirium, prior to them developing delirium. The identification of patients at risk would be useful, to institute earlier preventive measures, in order to prevent delirium. These interventions might include education, preparation, staff training around interventions (e.g. post‐operative mobilization), establishing a trustful relationship with the staff, and extending visiting times of loved ones and parents.

In this issue, three groups have tried to identify the risk factors of delirium: Habeeb‐Allah et al^9^ from Jordan, Gravante et al^10^ from Italy, Liang et al^11^ from Hong Kong. Habeeb‐Allah et al^9^ analysed risk factors in 245 patients after elective cardiac surgery, 9% being delirious, and identified advanced age and increased duration of surgery as risk factors. Several of these risk factors, such as the use of benzodiazepines, mechanical ventilation, severity of illness, and younger age are also identified as risk factors in PICU patients. Younger age as risk factor challenges the traditional assumption that age and delirium would have a linear relationship: the older the patient, the higher the risk for delirium. In fact, the relationship seems to be U‐shaped, with a high incidence in the early years, decreasing from 5 to 50 years, and then increasing again. These U‐shaped relationships require advanced statistical procedures, and common linear regression analysis might be misleading. Some factors such, as age, are fixed and cannot be changed. Other risk factors, such as surgery time, days on a ventilator, or sedatives/benzodiazepines, might be modifiable. These modifiable risk factors are a chance for nurses and other health care professionals to prevent delirium.

Gravante et al analysed 165 patients in two mixed ICUs, 56% being delirious, and found days in coma and severity of illness as risk factors, both seen as non‐modifiable. Whether days in coma is modifiable, is an interesting question. We would argue it might be, to some extent, e.g. by using frequent sedation assessment, targeted sedation, or bundles such as the ABCDEF (Assess, prevent, & manage pain; Both spontaneous awakening and spontaneous breathing trials; Choice of analgesia and sedation; Delirium: assess, prevent, and manage; Early mobility and exercise; Family engagement and empowerment) bundle.^12^ The authors used a tool for predicting the risk of delirium, the PRE‐DELIRIC (PREdiction of DELIRium in ICu patients), developed by Boogaard et al.^13^ This tool was evaluated by Liang et al in 375 mixed ICU patients with 44% being delirious. A higher PRE‐DELIRIC score was associated with the development of delirium, age, length of stay in the ICU, and mortality. Such an instrument is not yet available for children. The authors conclude that a higher score might help us to identify patients at risk and to start early preventive interventions, especially in light of reduced nursing resources. This may be correct, but that does not mean it is easy.

There is no single intervention that targets delirium alone. Recommended delirium‐preventing interventions such as early mobilization, family presence, and re‐orientation are multifaceted and have more goals than delirium prevention alone. Mobilization supports physical rehabilitation, weaning, and the chance of an earlier discharge; higher family presence improves well‐being, social interaction, coping, resilience; and re‐orientation helps to schedule activities of daily activities, participation in decision‐making,^14^ despite several challenges in time of Covid‐19,^15^ and especially in paediatric care.^16^ Hence, these interventions have more than one effect, with more than one purpose and implementing them only in patients at high risk might discriminate against those with a low risk of delirium. Moreover, withholding such interventions from patients with a low delirium risk might increase risk for other negative consequences, such as depression, loneliness, and reduced rehabilitation.

This leads to several questions. Identification of risk factors is one, but developing interventions in response to this is another. In particular, what should we do with high‐risk scores? Many delirium interventions disadvantage patients with a low risk, e.g. younger patients, with fewer co‐morbidities and shorter stay, giving them less rehabilitation, less family contact, less attention, because they have a lower risk. Our view is that prevention of delirium should not be seen as an isolated domain. It is intertwined with pain, sedation, distress, iatrogenic withdrawal syndrome, especially known in the paediatric setting,^17^ and/or other “triggering factors” towards a brain‐friendly environment and treatment.^18^ These features, such as pain, distress, and delirium can be accommodated under the umbrella of patients' *comfort*, and are part of the ABCDEF bundle. In adults, full compliance to this bundle showed significant and clinically meaningful improvements in outcomes like survival, mechanical ventilation use, coma, delirium, and post‐ICU discharge.^12^

It is well known there is no single magic intervention for optimizing *comfort* in paediatric and adult ICU patients, but multi‐component interventions work.^19^ In this issue, Sahawneh and Boss^20^ reviewed possible interventions to prevent and treat delirium for the adult population. It can be estimated that one third of delirium episodes might be preventable by good nursing care (arguably a better term than “non‐pharmacological intervention”). Yet, the evidence in critically ill children of the application of the full ABCDEF bundle performance is still lacking.^21^ Although early mobilization has been found safe and feasible in children, the effect on delirium prevention or treatment has not yet been established in children.

There are also promising results of interventions relating to increased family involvement, especially in children. In this issue, van den Hoogen et al^22^ showed how parents could be more engaged and integrated by using the values, opportunities, integration, control, and evaluation (VOICE) programme approach. Furthermore, in contrast to adults, a continuous family presence is normal in the PICU, and well accepted in most countries. However, there remains a difference between being present and being actively involved. Is holding someone's hand the same as re‐assuring that the other is safe and protected?

So how do we know that delirium interventions have been successful? Of course, we can collect data, such as number of assessments, calculating risk prediction models, counting time of delirium or even measure delirium severity. Success in delirium treatment, from a clinician's view, would be reducing delirium presence, days in delirium, and others. But what is important for patients or families, seeing their relatives or child crying for help? Other, patient‐ and family‐centred outcome parameters, such as reducing delirium burden, explaining, and understanding frightening experiences and life‐long coping, must be the next important measurements to prove our success. Success might be when a patient tells us: “Now I realize that they were bad dreams. Up until 3 days ago, all that stuff actually happened to me. Now I realize I was hallucinating.”^23^
